# An Improved Method for Extracting Viruses From Sediment: Detection of Far More Viruses in the Subseafloor Than Previously Reported

**DOI:** 10.3389/fmicb.2019.00878

**Published:** 2019-04-29

**Authors:** Donald Pan, Yuki Morono, Fumio Inagaki, Ken Takai

**Affiliations:** ^1^Department of Subsurface Geobiological Analysis and Research, Japan Agency for Marine-Earth Science and Technology, Yokosuka, Japan; ^2^Geomicrobiology Group, Kochi Institute for Core Sample Research, Japan Agency for Marine-Earth Science and Technology, Nankoku, Japan; ^3^Research and Development Center for Submarine Resources, Japan Agency for Marine-Earth Science and Technology, Yokosuka, Japan; ^4^Research and Development Center for Ocean Drilling Science, Japan Agency for Marine-Earth Science and Technology, Yokohama, Japan; ^5^Earth-Life Science Institute, Tokyo Institute of Technology, Tokyo, Japan

**Keywords:** virus, phage, subseafloor, sediment, subsurface, carbon, virus extraction, virus quantification

## Abstract

Viruses are the most abundant biological entities on Earth and perform essential ecological functions in aquatic environments by mediating biogeochemical cycling and lateral gene transfer. Cellular life as well as viruses have been found in deep subseafloor sediment. However, the study of deep sediment viruses has been hampered by the complexities involved in efficiently extracting viruses from a sediment matrix. Here, we developed a new method for the extraction of viruses from sediment based on density separation using a Nycodenz density step gradient. The density separation method resulted in up to 2 orders of magnitude greater recovery of viruses from diverse subseafloor sediments compared to conventional methods. The density separation method also showed more consistent performance between samples of different sediment lithology, whereas conventional virus extraction methods were highly inconsistent. Using this new method, we show that previously published virus counts have underestimated viral abundances by up to 2 orders of magnitude. These improvements suggest that the carbon contained within viral biomass in the subseafloor environment may potentially be revised upward to 0.8–3.7 Gt from current estimates of 0.2 Gt. The vastly improved recovery of viruses indicate that viruses represent a far larger pool of organic carbon in subseafloor environments than previously estimated.

## Introduction

Marine sediments are a major biome on Earth, covering ¾ of the Earth’s surface and containing approximately 10^29^ cells (Kallmeyer et al., 2012). Viruses are highly abundant within shallow marine sediments and have been observed in deep subsurface sediments as well (Bird et al., 2001; Middelboe et al., 2011; Engelhardt et al., 2014; Yanagawa et al., 2014). Viruses are the most abundant biological entities on Earth and play important biogeochemical and ecological roles in the ocean such as cell mortality and horizontal gene transfer (Suttle, 2005; Rohwer and Thurber, 2009). However, many questions remain regarding the roles of viruses in deep sediment. The progress of research on viruses in subseafloor sediment lags far behind that of subseafloor cellular life.

The major challenge in the study of subseafloor viral ecology is the technical problem of separating viruses from the surrounding sediment matrix. Methods exist for the separation and enumeration of viruses from sediments (Danovaro et al., 2001; Danovaro and Middelboe, 2010), however, the separation of viruses from sediment is complicated by interactions between viruses and particulate matter (Weinbauer et al., 2009). The efficiency of virus separation may vary with the composition of the sediment (Helton et al., 2006). Sediment properties such as mineralogy, organic content (Fuhs et al., 1985; Powelson et al., 1991), pH (Kinoshita et al., 1993; Loveland et al., 1996; Guan et al., 2003), as well as the isoelectric point of viruses (Dowd et al., 1998) can all influence the strength of virus-sediment adsorption. Clays (Lipson and Stotzky, 1983; Helton et al., 2006) and iron oxyhydroxide minerals are especially strong sorbents of viruses (Moore et al., 1981; Ryan et al., 2002; You et al., 2005; Zhu et al., 2005; Bradley et al., 2011; John et al., 2011).

Accurate virus quantification in sediment depends on techniques for the separation of viruses from sediment particles. Conventional virus extraction methods involve suspending the sediment in a buffer such as virus-free seawater (Danovaro and Middelboe, 2010), SM buffer (Yanagawa et al., 2014), or beef extract (Farkas et al., 2018), along with amendments like pyrophosphate (Danovaro et al., 2001), potassium citrate (Williamson et al., 2003), or EDTA (Helton et al., 2006) to aid in desorption. Conventional procedures typically include a shaking or sonication step to physically dissociate the viruses from sediment particles. The final step removes the sediment particles. In most conventional procedures, this involves pelleting the sediment by centrifugation in order to collect the viruses in the supernatant. Insufficient separation of viruses from sediment particles can result in underestimation of viral numbers or co-extraction of non-viral particles which obscures viruses and decreases their fluorescence (Montanié et al., 2015). Virus extraction efficiencies vary greatly between methods, and recovery can be as low as 0.09 to 0% in coastal and estuarine sediments (Miura et al., 2009; Hassard et al., 2016). The efficiency of conventional virus extraction techniques has not been assessed on a variety of sediment types, so comparisons of viral counts from different sediment lithologies may be problematic. Studies of cell extraction from subseafloor sediment show that cell recovery is also influenced by the sediment matrix, but improvements in cell separation methodology can improve recovery from across a variety of subseafloor sediment types (Morono et al., 2013). Improving the efficiency of separation from subsurface sediment may also make it possible to extract more viral DNA from less sediment, opening the doors to a wide spectrum of viral metagenomic studies in subseafloor sediments.

To this end, we developed a buoyant density based separation method modified from similar cell separation methods used for deep subseafloor sediments (Morono et al., 2013). The modified method utilizes a Nycodenz step gradient to separate sediment particles from virus extract. Using a variety of diverse subseafloor sediments, we compared the density separation method to conventional methods used for deep subseafloor virus enumeration (Engelhardt et al., 2014; Yanagawa et al., 2014).

## Materials and Methods

### Definitions

In this study, we infer SYBR-stained fluorescent particles that pass through some size cutoff, generally 0.2 μm, to be viruses. This operational definition will exclude prophage, giant viruses, and may exclude viruses that do not stain efficiently such as ssDNA and RNA viruses. It will also encompass membrane vesicles, gene transfer agents and very small cells. However, recent studies have suggested that membrane vesicles are only a minor portion of SYBR-stained particles compared to viruses (Biller et al., 2017). The majority of studies employing fluorescent staining techniques use this general criterion when determining values for “viral abundance” and “virus counts.” The term “virus-like particle” (VLP) has also been used to describe these particles, however, this can cause confusion because the term is also used in immunology to refer to virus-derived particles that lack nucleic acids. Even within the same study, the terms “VLP” and “virus” are often used interchangeably. Therefore, for the sake of consistency, we use only the term “virus,” inferred by SYBR-stained particles that pass through 0.2 μm filtration, throughout this paper.

Some studies of sedimentary viruses include a nuclease treatment step in order to reduce background fluorescence or to degrade extracellular DNA which may appear as fluorescing particles (Fischer et al., 2005; Helton et al., 2006; Danovaro and Middelboe, 2010). Because viral nucleic acids are protected by a capsid shell, nuclease treatment would not affect viruses. For the sake of comparison with previous subsurface virus studies (Engelhardt et al., 2014; Yanagawa et al., 2014), we have elected to not include a DNase I treatment step here because previous studies did not include this step. To test whether this was an acceptable omission, we conducted DNase I treatment on several samples and confirmed that DNase treatment did not make a substantial difference (Supplementary Figure S1), consistent with previous studies that have used DNase (Fischer et al., 2005; Danovaro and Middelboe, 2010).

### Density Based Virus-Sediment Separation

**FIGURE 1:**
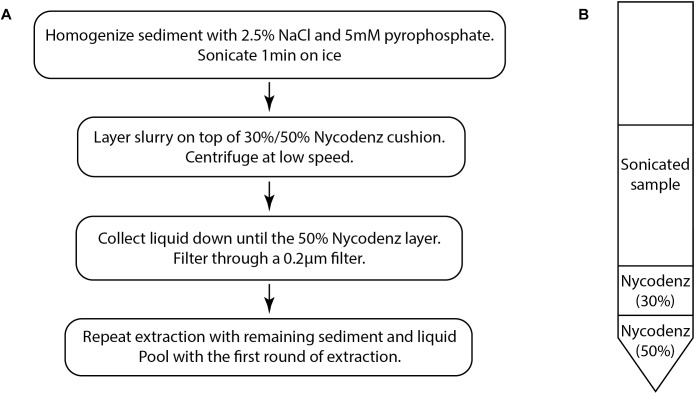
**(A)** The basic flow chart of the virus extraction protocol based on density separation. **(B)** Diagram of the density separation tube prior to centrifugation.

The density gradient separation method (Figure 1A) is adapted from previously published methods for virus separation (Danovaro and Middelboe, 2010) and cell separation from sediments (Morono et al., 2013).

All reagents were filtered through 0.02 μm Anotop syringe filters (GE Healthcare, 6809-2102) to remove viruses.

(1) Add a solution of 2.5% NaCl–5 mM sodium tetrapyrophosphate to 0.1–0.33 cm^3^ of sediment for a total volume of 6 mL. The volume of sediment that can be extracted will vary depending on the sediment characteristics. Some adjustments of volume may be necessary. See Section “Methodological Considerations.”

(2) Shake and vortex the mixture until solid clumps are broken apart and the sediment is dispersed into an even slurry. Then sonicate on ice for 1 min. We used a probe sonicator (15 W, UH-50 Ultrasonic Homogenizer SMT, Tokyo).

(3) Layer the sonicated sample onto a step gradient of 30 and 50% Nycodenz prepared in a 15 mL Falcon tube (Figure 1B). In this study, we used a volume of 1 mL for both Nycodenz layers. Centrifuge for 30 min at 2900 × *g* in a swinging bucket rotor. Low-speed centrifugation causes cells and sediment particles to migrate to denser layers while viruses should remain mostly unaffected. The majority of viruses should remain in the top layer, while a fraction of viruses will cross into the 30% layer, carried by the movement of larger particles. Theoretically, higher speed centrifugation may potentially be used, however, it was not tested due to limitations of the swinging bucket rotor used to develop this method.

(4) Using a syringe and needle, collect the liquid layers down to the 50% Nycodenz layer which may appear as a different color (Figure 1B). Care should be taken to avoid collecting the 50% layer because particles in this layer may obscure epifluorescence images. Filter the collected liquid through a 0.2 μm pore size PES (polyethersulfone) syringe filter (Merck Millipore SLGP033RS) in order to remove any remaining cells and large particulates. This step may also remove some large viruses.

(5) Further rounds of extraction can be performed in order to improve extraction efficiency. The number of times that a sample can be re-extracted may depend on the characteristics of the sediment. Add the NaCl-tetrapyrophosphate solution to the remaining material in the tube to a final volume of 6 mL and resuspend. Repeat the sonication and layer the suspension onto a fresh Nycodenz step gradient tube. After centrifugation, collect the liquid down to the 50% layer. Filter through a 0.2 μm pore size PES syringe filter.

(6) Pool the filtered extract from each round.

### Sediment Sample Collection and Description

Samples were selected to encompass a range of subseafloor sediment types. Nankai Trough samples representing clay rich sediments were collected during IODP Expedition 370 at Site C0023 Hole A located in the protothrust zone off of Cape Muroto, Japan (Heuer et al., 2017). Sediment cores were processed onboard and subsequently transported by helicopter to the Kochi Core Center (KCC) where subsamples were taken under cleanroom conditions and stored at -80°C (Heuer et al., 2017). Samples from offshore Shimokita, Japan, representing organic-rich hemipelagic sediments were collected from frozen cores collected during the *Chikyu* Shakedown Cruise CK06-06 at Site C9001 Hole C (Aoike, 2007). South Pacific Gyre samples representing organic poor, oligotrophic sediments were taken from frozen cores collected during IODP Expedition 329 at Sites U1366, U1367, and U1368 (D’Hondt et al., 2011, 2013, 2015). Iron mat sediment samples were collected from Tarama Knoll in the Okinawa Trough during cruise YK17-17 (Makita, 2017). Iron oxyhydroxide rich coastal sediment was collected from Nagahama Bay in Satsuma Iwo-Jima, Japan (Hoshino et al., 2015). These iron-rich samples were stored at 4°C.

### Comparison of Methods

#### Re-analysis of Previously Published Virus Counts From Subseafloor Sediment

Using subseafloor sediment samples for which virus counts had been previously published, virus enumeration was repeated using the density separation method. Enumeration was repeated on samples from the South Pacific Gyre [International Ocean Drilling Program (IODP) Exp. 329] (Engelhardt et al., 2014) and offshore Shimokita Peninsula (Chikyu Cruise CK06-06) (Yanagawa et al., 2014), representing oligotrophic sediments and organic-rich hemipelagic sediments, respectively. Viruses were enumerated from two rounds of extraction, however, only one round of extraction was possible for the Shimokita samples due to clogging of Anodisc filters. In order to calculate the virus-to-cell ratio (VCR), cell counts were obtained from previously published data for the same core samples/depths (Engelhardt et al., 2014; Yanagawa et al., 2014; D’Hondt et al., 2015). Values obtained using the density separation method were compared to the previously published values. For two samples, published virus counts did not exist, so viruses were re-extracted and enumerated following the methods used in the original publications.

#### Method Comparison for Iron-Rich Sediment Samples

Samples of iron mat from Tarama Knoll, Okinawa Trough, Japan (Makita, 2017) and iron-rich sediment from Nagahama Bay, Satsuma, Japan (Hoshino et al., 2015) were also used to demonstrate the effectiveness of the density separation technique for extracting viruses from sediment containing high concentrations of iron oxyhydroxides. Viruses were enumerated from two rounds of extraction. Results were compared to virus counts obtained by the conventional virus separation method used by Engelhardt et al. (2014).

#### Evaluation of Recovery Efficiency

The recovery efficiency of the density separation method was evaluated using sediments from various depths from the Nankai Trough (Site C0023 Hole A), representing several sedimentary units and lithologies from 257 to 829 m below the seafloor (mbsf) (Heuer et al., 2017). A known concentration of viruses collected from seawater via filtration (0.2 μm) was amended to 0.33 cm^3^ of sediment and allowed to adsorb for 15 min. We verified (using the density separation method) that the abundance of indigenous viruses in the sediment was insignificant (less than 1 per field of view at 1000× magnification) compared to the number of viruses applied. The viruses were then extracted (two rounds) using the density separation protocol described above. The extraction was also performed using conventional protocols used for virus counts of subseafloor sediment cores (Engelhardt et al., 2014; Yanagawa et al., 2014). To summarize briefly, in the protocol employed by Engelhardt et al. (2014), 1 cm^3^ of sediment is added to 3.5 mL ddH_2_O and 1 mL sodium pyrophosphate (55 mM) and turned into a slurry. The slurry is mixed for 15 min and sonicated. The sediment is then pelleted. The supernatant is retained, and Tris-EDTA buffer is used to wash the pellet in order to retrieve more viruses. In the protocol employed by Yanagawa et al. (2014), 3 cm^3^ of sediment is added to 10 mL of SM buffer containing 2% formaldehyde. After mixing the slurry, the sediment is sonicated. The sediment is then pelleted, and the supernatant is retained. In order to make a comparison of the methods, we adjusted all volumes for 0.33 cm^3^ of sediment and used the same filtration and staining steps as the density separation method.

To determine the number of rounds of extraction necessary to maximize the recovery of viruses from a single sample, repeated extractions were performed. A known concentration of viruses collected from seawater via filtration (0.2 μm) was spiked to 0.33 cm^3^ sediment samples from the Nankai Trough (Heuer et al., 2017) and South Pacific Gyre (Engelhardt et al., 2014) and allowed to adsorb for 15 min. Viruses were extracted for five iterations and enumerated after each round.

#### Enumeration of Viruses

Viruses were enumerated by epifluoresence microscopy based on standard methods (Suttle and Fuhrman, 2010). In brief, separated viruses were filtered onto 0.02 μm Anodisc filters (GE Healthcare, 6809-6002) and stained by SYBR Green I (Invitrogen, S7567) for 15 min in the dark according to standard procedures. Excess stain was removed by vacuum filtration. Filters were mounted onto slides with VECTASHIELD antifade mounting medium (Vector Laboratories, H-1000) and enumerated under epifluorescence at 1000× magnification (Olympus BX53 microscope with 130 W U-HGLGPS fluorescence light source, Olympus U-FGFP filter cube). Fluorescent beads (Fluoresbrite BB Carboxylate Microspheres 0.5 μm, Polysciences, 18339-10) were applied to filters when it was necessary to help with focusing. Images of random fields were captured by camera connected to a computer and enumerated to determine the original concentration of viruses.

## Results and Discussion

### Deep Subseafloor Sediments

**FIGURE 2:**
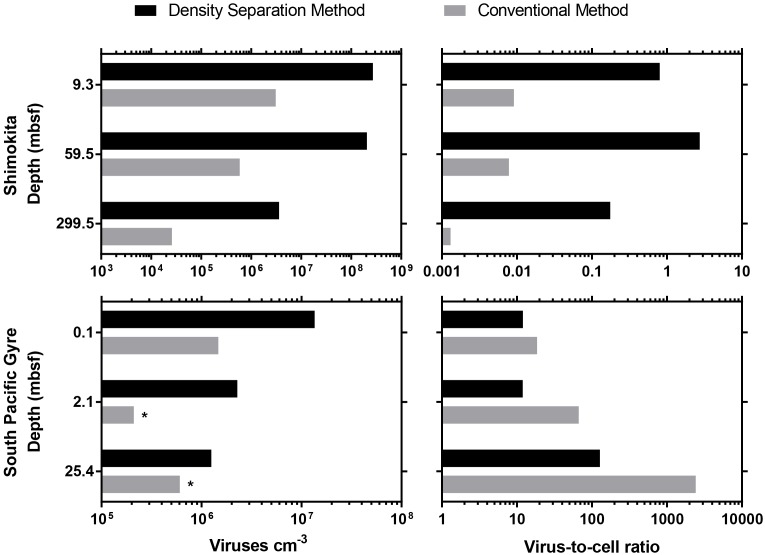
Comparison between the density separation method and conventional methods used in previously published viral enumeration studies from IODP subseafloor sediment cores. ^∗^indicates a sample that did not have a previously published result, so the sample was re-enumerated according to the original published method. For Shimokita samples, only a single round of extraction was possible.

Previously published studies of deep subseafloor sediment viruses (Engelhardt et al., 2014; Yanagawa et al., 2014) were re-evaluated using the new density separation method. The density separation method resulted in dramatically greater numbers of extractable viruses. Using the new method, 2–10 times more viruses were extracted from South Pacific Gyre samples compared to the originally published results, whereas 87–350 times more viruses were extracted from Shimokita sediments (Figure 2). Accordingly, the VCR of these Shimokita samples, originally reported as less than 0.01, now have a range of 0.17–2.7, altering how such data would be interpreted. While the VCR of South Pacific Gyre samples should also increase along with the virus counts, the VCR decreased due to higher cell abundances in revised cell counts (D’Hondt et al., 2015). Despite this, the updated VCR we calculated were in basic agreement with the trends and interpretations of the original publication (Engelhardt et al., 2014).

Sediment organic matter may cause high background fluorescence that obscures the epifluorescence imaging of viruses. South Pacific Gyre sediments are characterized as metalliferous and organic-poor (0.25% TOC to below detection) (Aoike, 2007; D’Hondt et al., 2015), whereas samples from offshore Shimokita are high in organic matter (avg. 1.35%) (Kobayashi et al., 2008). During the analysis of viruses extracted from Shimokita samples, we encountered high background noise which obscured the imaging of viruses, so we found it necessary to lower the volume of sediment (0.1 cm^3^) that we could extract and also to limit the extraction to a single round. We experienced clogged Anodisc filters or poor visibility when working with higher sediment volumes (0.2 cm^3^) or multiple rounds of extraction. Because of this, the high amounts of sediment organic matter in the Shimokita sediment may have caused previous attempts to enumerate viruses to result in 2 orders of magnitude fewer viruses than what we found by the density separation method.

### Iron-Rich Sediments

**FIGURE 3:**
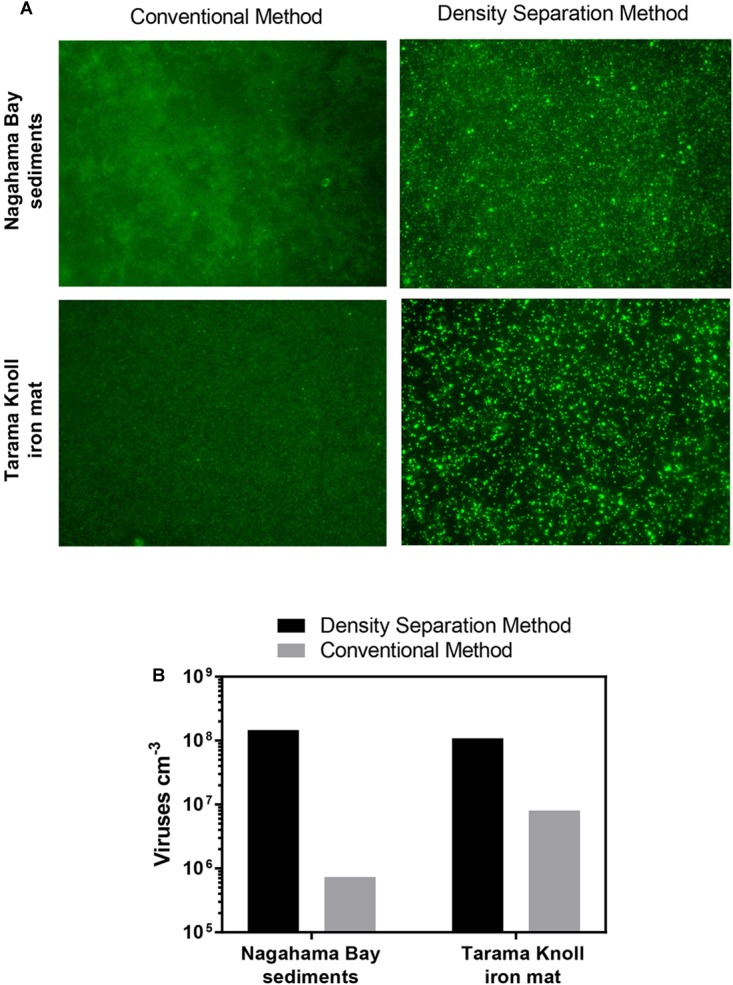
**(A)** Fluorescence images of SYBR Green I stained viruses extracted using the density separation method and conventional method. The density separation method dramatically improves the separation and fluorescence of viruses from iron rich sediment. Iron oxyhydroxide floc from Nagahama bay, Satsuma and an iron mat sample from Tarama Knoll, Okinawa Trough was used. **(B)** Comparison of the virus counts resulting from the density separation method and conventional method. Viral abundance for the density separation method may be underestimated due to the presence of some overlapping fluorescent particles in the micrographs.

Iron oxyhydroxide minerals are strong sorbents of viruses (Moore et al., 1981; Ryan et al., 2002; Bradley et al., 2011). The strong affinity between iron oxyhydroxide minerals and viruses has been utilized in industrial applications to remove viruses from wastewater (You et al., 2005; Zhu et al., 2005) as well as scavenge and concentrate viruses from large volumes of water (John et al., 2011). Because the presence of iron oxyhydroxide minerals within sediments may pose a problem for virus separation, we compared the effectiveness of the density separation method to the conventional method using iron rich sediment from Nagahama Bay (Hoshino et al., 2015) and an iron mat sample from Tarama Knoll, Okinawa Trough, Japan (Makita, 2017). In both samples tested, the density separation method resulted in higher viral abundances by 1–2 orders of magnitude. The density separation method increased viral abundance by 200-fold in the Nagahama Bay sample, from 1.81 × 10^6^ viruses/cm^3^ to 3.61 × 10^8^ viruses/cm^3^ (Figure 3B). In the iron mat sample, viral abundance was increased 13-fold to 1.08 × 10^8^ viruses/cm^3^. The ratio of the fluorescence signal of the viruses to background noise was greatly improved compared to the conventional method due to a better separation of high-density abiotic particles by the density separation method (Figure 3A). This was observed as significantly less particle accumulation on the Anodisc filters (Supplementary Figure S2). The use of density layers may also help to improve virus extraction efficiency by acting as a barrier between the separated viruses and iron oxyhydroxide particles, preventing re-adsorption. Conventional methods do not have any barrier between viruses and the pelleted sediment particles, so there is a greater opportunity for viruses to re-adsorb to the highly adsorptive iron minerals.

### Virus Recovery Efficiency

**FIGURE 4:**
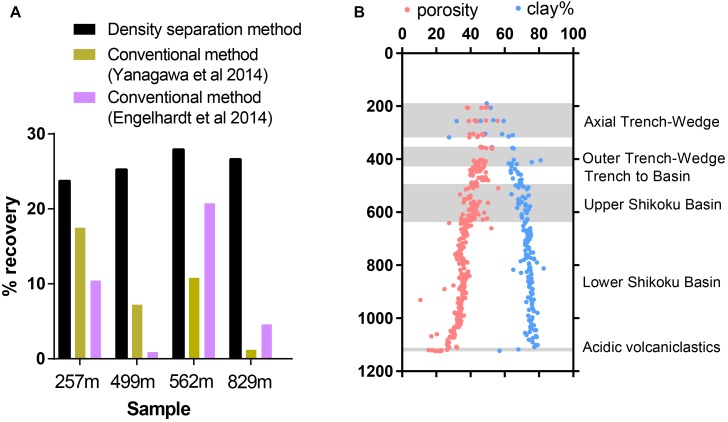
**(A)** Efficiency of extraction of spiked viruses from sediment from Nankai Trough, Japan. **(B)** Sediments represent various sedimentary units with a range of porosities and clay content. Shadowed areas highlight the locations of alternating sedimentary units. Porosity and clay data reproduced from IODP Expedition 370 Report Summary (Heuer et al., 2017).

Within a single sediment core, sediment lithology may vary dramatically. Previous studies have shown that sediment physical and chemical properties can influence the extractability of viruses from sediment (Helton et al., 2006), so we tested virus recovery efficiency on a single sediment core covering several sedimentary units with varying lithological features, porosity, and clay content. The efficiency of virus recovery was tested by spiking a known concentration of seawater viruses to sediment samples collected at various depths from the Nankai Trough Site C0023 (257–829 mbsf). The sediments chosen for this test represent various different sedimentary units with a range of porosities and clay content, while organic carbon ranged from 0.2 to 0.5% TOC (Heuer et al., 2017). The density separation method was able to consistently recover between 23.5 and 27.9% of the spiked viruses whereas the conventional methods varied greatly between samples (Figure 4A). Using the conventional method employed by Yanagawa et al. (2014), virus recovery varied from 17.3% at the Axial Trench-Wedge (sand, silt, and mud, 257 mbsf) to 1.2% at the Lower Shikoku basin (hemipelagic mudstone, 829 mbsf). The recovery efficiency of the method used by Engelhardt et al. (2014) varied from as high as 20.7% in the Upper Shikoku Basin (562 mbsf) to as low as 0.88% for sediment between the Trench-to-Basin and Upper Shikoku Basin units (499 mbsf) (Figure 4B). This large variability in virus extraction efficiency when using conventional methods can explain the large variabilities in viral abundance observed in previously published sediment core depth profiles (Yanagawa et al., 2014; Peduzzi, 2016) and other sediment samples (Pinto et al., 2013). These data show that conventional methods do not have the consistency necessary for confident comparisons of viral abundances within depth and lithological profiles. This suggests that the high variability in sediment virus abundances observed in previous publications may be more reflective of the efficiency of virus extraction rather than actual abundances. Even between the two conventional methods, the recovery efficiency differed greatly. The reason may be due to the use of different buffers, differences in the proportion of sediment to buffer as well as sonication times and the number of rounds of extraction. Using conventional virus extraction methods, the large variability in extraction efficiency makes it challenging to make meaningful comparisons between datasets from different studies or even different sites/depths within the same study (Pinto et al., 2013).

Nankai Trough sediments consist of high proportions of clay minerals, which can cause inefficient virus extraction due to the high adsorptivity of viruses onto clays (Sobsey et al., 1980; Chattopadhyay and Puls, 1999; Helton et al., 2006). The strength of adsorption between viruses and clays is positively correlated to the cation exchange capacity of clay minerals (Lipson and Stotzky, 1983). The primary clay mineral is smectite, with illite becoming the primary clay in the deeper portion of the profile. Even though smectite has a higher cation exchange capacity than illite, the consistency of the density separation method is maintained through the smectite-illite clay mineral transition in the profile (Moore et al., 2005; Heuer et al., 2017).

**FIGURE 5:**
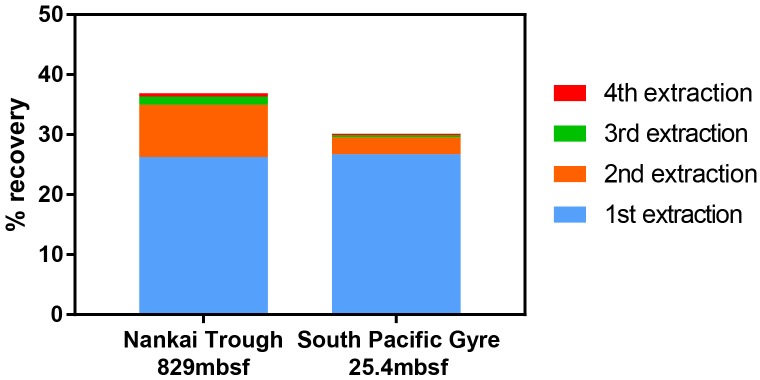
Recovery of spiked viruses from sediment with sequential extractions. Two extractions were sufficient to recover nearly all of the viruses that could be extracted. A fifth extraction was performed, but the contribution to the total virus count was negligible.

Furthermore, when using the conventional virus separation approach, as many as 30–50 repeated extractions may be needed to obtain the maximal number of viruses (Siem-Jørgensen et al., 2008). With the low extraction efficiency of conventional extraction approaches, each extraction may result in the removal of only a small fraction of viruses such that a large number of extractions would be required to approach the maximal number. Because conducting 30–50 serial extractions is impractical, extraction procedures are often limited to 1–3 extractions. With the density separation method, only 2 rounds of extractions are sufficient to extract close to the total number of viruses that would be extracted by further rounds (Figure 5).

### Methodological Considerations

Extraction of spiked viruses from the sediments shows that only 20–40% of viruses were able to be recovered despite multiple extractions. It is possible that a fraction of the spiked viruses may be destroyed upon contact with certain minerals in the sediment (Ryan et al., 2002; Harvey and Ryan, 2004; Pecson et al., 2012). This may mean that a 100% recovery of all spiked viruses may not be possible. If this is not the primary reason for the relatively low recovery, the remaining unextractable viruses may be irreversibly bound to the sediment or lost during the extraction process. It may be possible to push the recovery closer toward 100% with improved methods. For now, the density separation method is the most effective method for virus extraction from subseafloor sediments (Supplementary Figure S3).

Oversonication may also destroy viruses, so optimization of the sonication procedure may help to improve recovery (Danovaro et al., 2001). The optimal strength and length of sonication will depend on the sonicator model and tip diameter as well as the sediment characteristics. Sonication has sometimes been found to be problematic enough to warrant omission from some virus extraction methods (Montanié et al., 2015). Experimenters are encouraged to find the optimal settings for their own sonication system.

The volume of sediment that can be extracted in a single extraction should be adjusted according to the sediment characteristics. In the case of organic rich sediments, the amount of extract that can be filtered through a single Anodisc filter may be limited, likely due to incomplete separation of viruses from other low-density organic particles in the 20–200 nm size range. Organic-rich sediments such as the Shimokita samples had to be limited to 0.1 cm^3^. Attempts at filtering extracts from larger volumes (0.2 cm^3^) of Shimokita sediment through Anodisc filters resulted in clogged and unusable filters. For low organic sediments from the South Pacific Gyre and Nankai Trough, we did not encounter clogging of Anodisc filters. Pooling multiple extracts from high organic Shimokita sediments also ran into the same problem. In such cases, it would be necessary to use separate Anodisc filters for each round of extraction, which may be cost prohibitive. Samples high in organic matter [e.g., terrestrial soils (Williamson et al., 2013; Trubl et al., 2016)] may require some method optimization in order to further separate viruses from other organic particles.

In our virus extraction method, a final step of filtration (0.2 μm) is used to separate cells and large particulates from viruses. Due to this cutoff, giant viruses will be excluded. Some small fraction of viruses that are smaller than 0.2 μm in diameter may potentially also be caught onto the filter as well. It may be possible to use larger size cutoffs or even forego final filtration. However, forgoing filtration may come with the risk of introducing some cells or large organic particulates into the extraction. If the presence of small cells is a major concern, a 0.1 μm final cutoff may be used instead, however, virus abundances may potentially decrease. In any case, the experimenter should determine what works best for their research goals.

Viruses extracted using the density separation technique may potentially retain their infectivity, allowing for isolation and culture-based applications. Viruses that are extracted from sediment minerals may still retain their infectivity (Schaub and Sagik, 1975). Clay minerals such as montmorillonite, attapulgite, vermiculite, and kaolinite have been demonstrated to protect viruses from decay or inactivation (Bitton and Mitchell, 1974; Babich and Stotzky, 1980). Because Nycodenz has been successfully applied to the isolation of viable cells from other materials (Hevia et al., 2015), it is plausible that the viruses extracted by the density separation method may also be viable, however, this has not been verified yet. If the use of Nycodenz as the compound to generate the density layers does not yield viable viruses, other compounds may potentially be used. Other compounds such as polytungstate have been successfully used to separate cells from sediment (Morono et al., 2013).

### Implications

There is estimated to be on the order of 10^31^ viruses on Earth, the overwhelming majority of which inhabit soil and sediments, with estimates ranging from 79% (Bar-On et al., 2018) to 97% (Cobián Güemes et al., 2016). By some estimates, viruses in marine subseafloor sediments are estimated to comprise approximately half of the viruses on Earth (4.8 × 10^30^) (Bar-On et al., 2018). The density separation method has demonstrated that conventional methods underestimate sediment viral abundance and that viruses may in fact be far more numerous than previous studies have indicated. Although the total abundance of marine sediment viruses is not yet well constrained, we can attempt to estimate the new method’s potential for revising current estimates of the pool of viral carbon in subseafloor sediments. We evaluate the potential based on the factor increase we observed when employing the density separation method and applying the factor increases to current estimates of subseafloor viral biomass (Bar-On et al., 2018). Oligotrophic, open ocean sediments such as those from the South Pacific Gyre cover 42% of the ocean while containing 10% of the cells (Kallmeyer et al., 2012). Within the South Pacific Gyre sediments, we observed an increase of a factor of 2–10 when we applied the density separation method. Coastal sediments such as those from Shimokita cover 7% of the ocean but carry 33% of the cells in subseafloor sediment. Using Shimokita sediments to represent coastal sediment, we observed an increase of a factor of 80–350 when we use the density separation method. For the remaining portion of the ocean, we will use a conservative factor of 1–10 for the possible increase in virus abundance that may possibly be expected. Using these correction factors, the total abundance of subseafloor viruses may potentially be revised upward to 3.3–16.2 × 10^31^ viruses. This would result in revised estimates of 0.8–3.7 Gt of viral carbon in the deep subsurface compared to the current estimate of 0.2 Gt (Bar-On et al., 2018). The improved recovery over conventional methods indicates that a large portion of viruses have heretofore been undetected in subsurface sediment, suggesting that the global subsurface biomass of viruses may be far larger than previously estimated. Revising viral abundance numbers for subseafloor sediments may significantly alter the subseafloor carbon budget, especially if the viral pool of carbon approaches the same order of magnitude as the prokaryotic pool (approximately 10 Gt C) (Bar-On et al., 2018). Revised estimates of viral biomass indicate that viruses may actually an important pool of subsurface organic matter, especially organic phosphorus (Jover et al., 2014), for supporting subseafloor microbial populations (Dell’Anno et al., 2015).

Ratios of viruses to cells have been used to interpret microbial ecology (Wigington et al., 2016; Parikka et al., 2017), however, previously published VCR values measured by conventional methods from sediment must be reconsidered in light our findings. For example, VCR observed in environments such as seafloor chimney structures [as low as 0.002 (Yoshida-Takashima et al., 2012)] may need reinterpretation if the density separation method dramatically increases viral extraction efficiency. We have shown that conventional methods can vary greatly in extraction efficiency between samples of different lithology, even within a single sediment core (Figure 4A). We suggest that VCR measured by conventional sediment extraction methods should not be the basis of comparison between different sedimentary environments unless if there is reason to believe that the extraction efficiencies would be expected to be similar, such as two samples very close in proximity and composition. It is especially not recommended to compare VCR between solid-associated and aquatic samples due to methodological differences. Researchers must also consider differences between virus extraction efficiency and cell extraction efficiency when interpreting results, especially if the methodology used for the extraction of microbial cells and viruses differs substantially (Parikka et al., 2017). Because the density separation method is methodologically based on a similar density-based cell extraction method (Morono et al., 2013), it is ideal to pair these two methods for calculation of VCR in sediment.

While recent advances in cell-sediment separation techniques have allowed cells to be quantified at extremely low biomass (<10 cells/cm^3^) (Morono et al., 2013, 2017; Inagaki et al., 2015; Morono and Inagaki, 2016), virus-sediment separation techniques have not made similar advances. The detection limits of conventional sediment virus separation and enumeration techniques have already been reached for some sites such as the South Pacific Gyre (Engelhardt et al., 2014). As IODP and ICDP expeditions begin to investigate subsurface life in sediments with conditions approaching life’s limits, the development of the density separation method makes it possible to study viruses in deeper, lower biomass environments than ever before.

## Author Contributions

DP conceived, designed and conducted all experiments, and analyzed the data. YM contributed to method development. DP wrote the manuscript with contribution from YM, FI, and KT.

## Conflict of Interest Statement

The authors declare that the research was conducted in the absence of any commercial or financial relationships that could be construed as a potential conflict of interest.
