# Supplementary material for: An Improved Method for Extracting Viruses From Sediment: Detection of Far More Viruses in the Subseafloor Than Previously Reported
Source: Front Microbiol. 2019 Apr 29;10:878. doi: 10.3389/fmicb.2019.00878 (PMC6501758; doi:10.3389/fmicb.2019.00878)
Supplement: Supplementary file 1 [file Data_Sheet_1.pdf]

Supplementary Material

An Improved Method for Extracting Viruses From Sediment: Detection of Far More Viruses in the Subseafloor Than Previously Reported

Donald Pan[1†*], Yuki Morono[2,3†], Fumio Inagaki[2,3,4‡], Ken Takai[1,3,5†]

[1]Department of Subsurface Geobiological Analysis & Research, Japan Agency for Marine-Earth Science and Technology (JAMSTEC), Yokosuka, Kanagawa, Japan

[2]Geomicrobiology Group, Kochi Institute for Core Sample Research, JAMSTEC, Nankoku, Kochi, Japan

[3]Research and Development Center for Submarine Resources, JAMSTEC, Yokosuka, Kanagawa, Japan

[4]Research and Development Center for Ocean Drilling Science, JAMSTEC, Yokohama, Kanagawa, Japan

[5]Earth-Life Science Institute, Tokyo Institute of Technology, Meguro, Tokyo, Japan

[†]Present address: X-STAR, Japan Agency for Marine-Earth Science and Technology (JAMSTEC), 2-15 Natsushima-cho, Yokosuka, Kanagawa, 237-0061, Japan

[‡]Present address: Mantle Drilling Promotion Office (MDP), Institute for Marine-Earth Exploration and Engineering (MarE3), Japan Agency for Marine-Earth Science and Technology (JAMSTEC), Showa-machi 3173-25, Kanazawa-ku, Yokohama, Kanagawa, 236-0001, Japan

*** Correspondence:**
Corresponding Author
donald.pan@jamstec.go.jp

Supplementary Figures

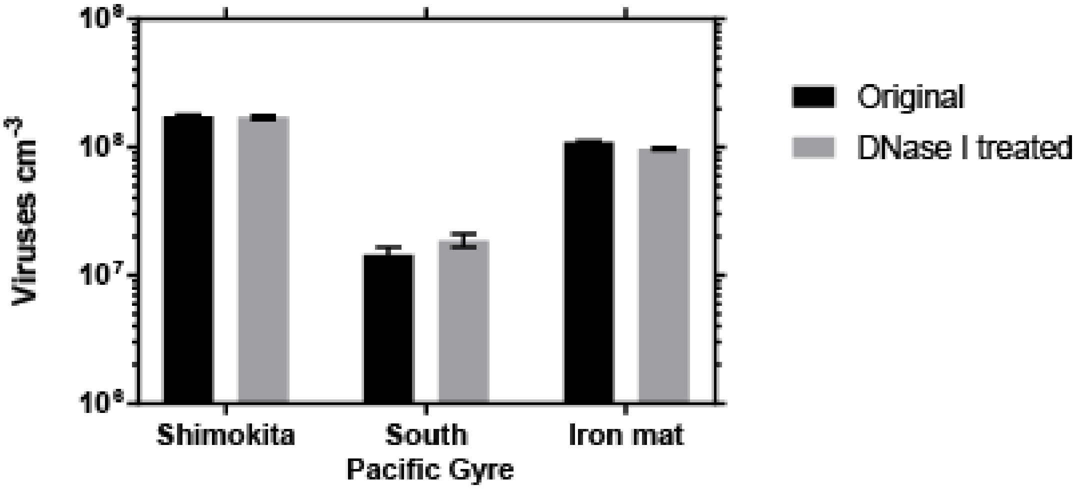

Supplementary Figure S1. Comparison of virus counts by the density separation method with and without a DNase I step to degrade extracellular DNA. DNase I (0.5 U mL^{-1} final concentration) was added to the solution of viruses following separation according to Danovaro & Middelboe (2015). Samples were from offshore Shimokita, South Pacific Gyre, and an iron mat from Tarama Knoll, Okinawa Trough. Error bars represent the 95% confidence interval for the estimate of viral abundance.

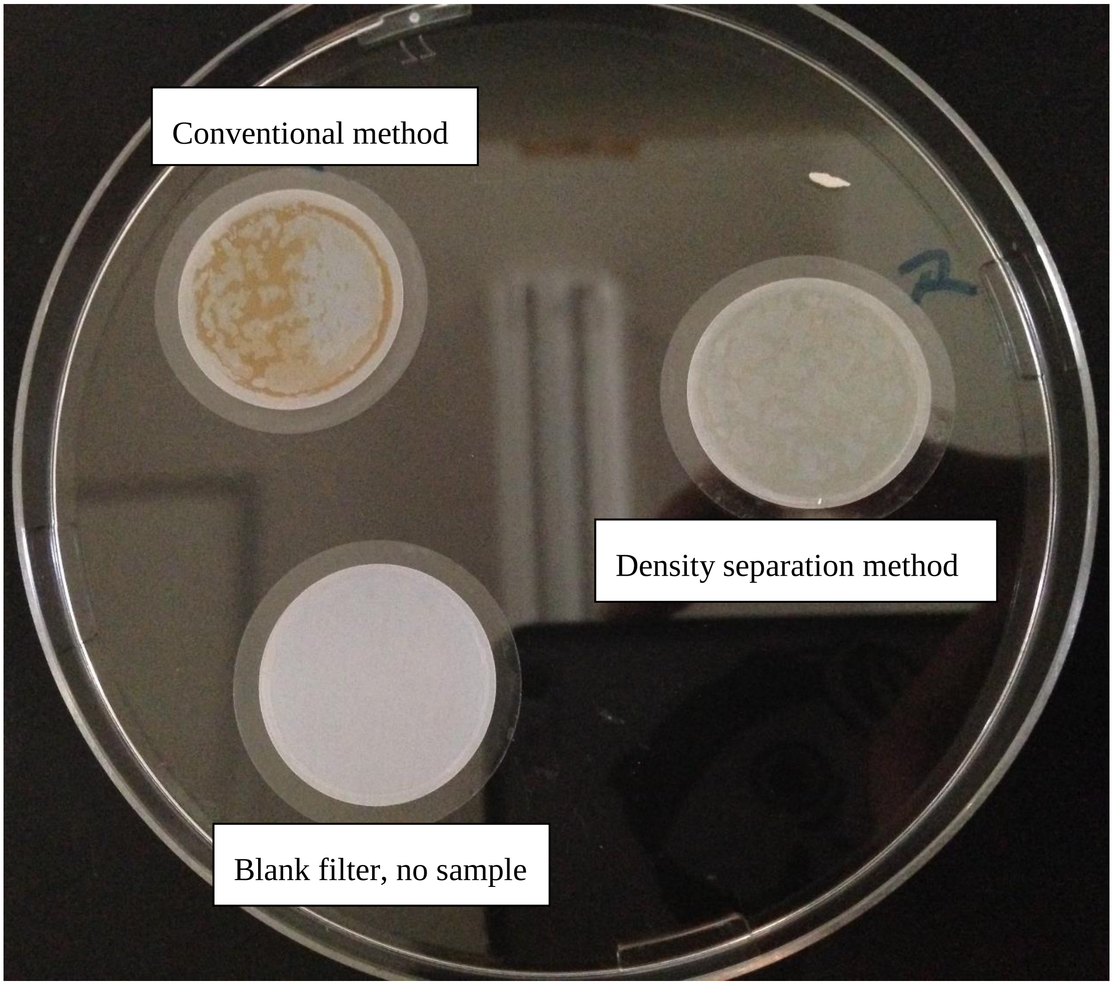

Supplementary Figure S2. More particulate particles are removed by the density separation method compared to the conventional method. Many particulates (shown by the orange-brown color) remain after conventional virus separation. A blank filter is shown for comparison. The sample used here was from an iron oxide floc (Nagahama Bay, Kagoshima).

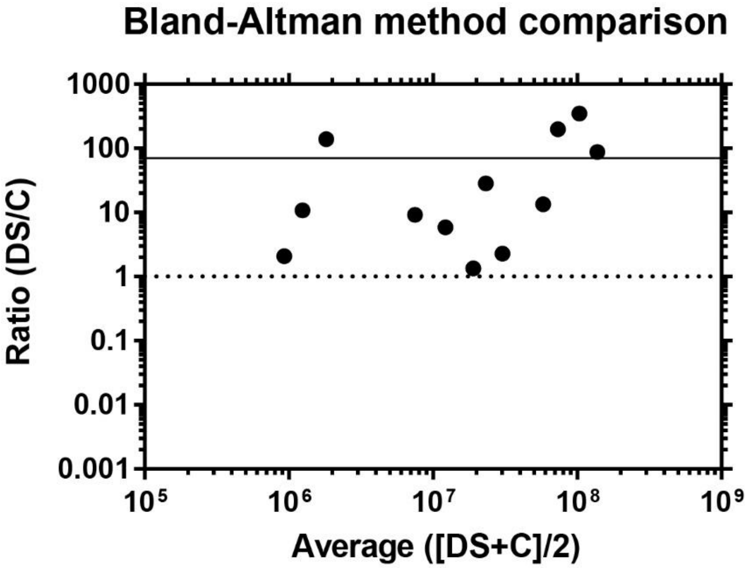

Supplementary Figure S3. Bland-Altman plot comparing the results of virus counts between the density separation (DS) method and conventional (C) method. Data shown in this plot are derived from virus counts measured from all sediment samples presented in this study. The protocol employed by Engelhardt and colleagues (2014) was used for the conventional method. The dashed line indicates a 1:1 correspondence between the DS and C methods. The solid line shows the average factor improvement(70X) of the DS method over the C method.
